# Accuracy of Inferences About the Reproductive Number and Superspreading Potential of SARS-CoV-2 with Incomplete Contact Tracing Data

**DOI:** 10.21203/rs.3.rs-3760127/v1

**Published:** 2023-12-29

**Authors:** Henry Bayly, Winnie Mei, Debra Egeren, Madison Stoddard, Arijit Chakravarty, Laura F White

**Affiliations:** Boston University School of Public Health; University of Washington School of Public Health; Stanford University; Fractal Therapeutics; Fractal Therapeutics; Boston University School of Public Health

## Abstract

The basic reproductive number (R_0_) and superspreading potential (k) are key epidemiological parameters that inform our understanding of a disease’s transmission. Often these values are estimated using the data obtained from contact tracing studies. Here we performed a simulation study to understand how incomplete data due to preferential contact tracing impacted the accuracy and inferences about the transmission of SARS-CoV-2. Our results indicate that as the number of positive contacts traced decreases, our estimates of R_0_ tend to decrease and our estimates of *k*tend to increase. Notably, when there are large amounts of positive contacts missed in the tracing process, we can conclude that there is no indication of superspreading even if we know there is. The results of this study highlight the need for a unified public health response to transmissible diseases.

## Introduction

Two key epidemiological parameters that inform our understanding of the transmission dynamics of infectious diseases, in particular infectious respiratory diseases, are the basic reproductive number and superspreading potential, estimated by the overdispersion parameter [1]. Several methods to estimate the reproductive number have been developed, including using dynamic mathematical models [2, 3], statistical methods using case notification data [4, 5], and data from contact tracing studies with directly observed transmission events [6–8]. Superspreading is an important feature of an infectious disease and is often not well-quantified. The impact of superspreading can be substantial in designing interventions, modeling studies, and clinical studies to test therapeutics [9]. Methods to estimate superspreading are not as well-developed and typically have relied heavily on results from contact tracing investigations, though increasingly analytical approaches to this problem are being developed [10, 11]. In particular, early in the SARS-CoV-2 pandemic contact tracing information was the primary source of information on transmission dynamics, in particular superspreading behavior [12–14].

While contact tracing studies are important to understanding the epidemiology of infectious diseases, their limitations are also well-documented. This is especially apparent in household contact studies, one of the more common contact tracing study designs. In influenza and tuberculosis (TB), household designs for understanding transmission dynamics and infection control are common [15–19]. However, there are numerous studies indicating that extra-household transmission is important and substantial for both diseases [17, 19–23], limiting the interpretability of household-based contact tracing both in making inferences about transmission parameters and as a tool for infection control.

More recently, early in the COVID-19 pandemic, contact tracing, including extra-household contacts, was widely used in an attempt to both contain disease transmission and estimate the reproductive number and overdispersion parameter for SARS-CoV-2 [12, 24, 25]. This proved to be relatively successful for controlling transmission when coupled with other control measures in many East Asian countries [26–30] and in some university settings [31]. However, contact tracing was relatively ineffective in the general population of the United States [32–34]. For instance, one study during the COVID-19 pandemic in the United States found that 68% of positive index cases reported having 0 contacts, undermining the reliability of this approach [35]. In a previous work, we combined data from multiple contact tracing studies using a Markov model, to estimate that in the United States, the chance of identifying a positive contact of a positive index case was about 1.65% (assuming the use of PCR tests) [36]. In addition to the clear problems this poses for infection control, this incompleteness in basic contact tracing data leads to a question of just how accurate our inferences about the transmission dynamics of SARS-CoV-2 really are. Further, it is unclear how generalizable inferences are on transmission parameters obtained in settings with extensive containment measures in place, such as those implemented in many Asian countries, where transmission opportunities outside of a household were extremely limited.

Early on in 2020 there were numerous studies done to estimate the values of the basic reproductive number, $R_{0}$, and overdispersion parameter, k. Many of these studies came out of East Asian countries, where contact tracing policies were stringent with high levels of adherence. Summaries of these early studies found that $R_{0}$ generally ranged between around 1.5 and 6.5 [37, 38]. One of the earliest estimates of k came from contact tracing data from Hong Kong and estimated k to be 0.43 (95% CI: 0.29–0.67), indicating the presence of significant superspreading [39]. The ability to generalize these results to other settings is difficult due to the variability in control measures and modifications in behavior implemented worldwide. Results obtained from countries with stricter contact tracing measures likely yielded more complete data. However, the act of doing stricter contact tracing likely correlates with a reduction in spread potentially underestimating the transmission potential of the virus.

Here we investigated the accuracy of our inferences about the basic reproductive number and superspreading potential of SARS-CoV-2 in the face of incomplete contact tracing data. Specifically we focus our attention to incomplete data with bias due to preferential contact tracing within households.

## Methods

### Statistical Model

We use the basic reproductive number R0 and overdispersion parameter k to describe the characteristics of SARS-CoV-2 transmission. R0 and k are estimated by the mean and the dispersion parameter of a negative binomial (NB) distribution fitted to the secondary infection distribution. The probability mass function of the negative binomial distribution using parameterization of p, the probability of success, and n, the number of trials, is given by:

(1) $$P(X=x;n,p)=\frac{\Gamma (x+n)}{\Gamma (x+1)\Gamma (n)}p^{n}(1-p{)}^{x}$$

To estimate $R_{0}$ and k, this can be reparametrized, as shown in Lloyd Smith [40]:

(2) $$P=\frac{\Gamma (x+1/\alpha )}{\Gamma (x+I)\Gamma (1/\alpha )}{\left(\frac{I}{I+\alpha \mu }\right)}^{1/\alpha }{\left(\frac{\alpha \mu }{I+\alpha \mu }\right)}^{x}$$

where k=l/α and $R_{0}=\mu$. A k value of less than 1 suggests overdispersion (in this case superspreading behavior). Here x represents the number of secondary infections from a single index case.

### Simulation model

In order to generate contact tracing data to form a secondary infection distribution we used Covasim, a stochastic agent-based simulator for performing COVID-19 analyses [41]. Covasim allows the user to customize numerous parameters that dictate how SARS-CoV-2 spreads across a target population.

We consider the impact of preferential contact tracing, specifically the scenario where contact tracing is predominantly identifying household contacts. We utilized Covasim’s hybrid layer network type which defines four unique layers (Home, Work, School, and Community) where an agent potentially has contacts. We altered the parameters of the negative binomial distribution used to create the secondary transmission chain to produce scenarios with and without superspreading. Additionally, we altered the transmission probability of the household layer to create two cases: one where there was higher in-home transmission relative to the other layers and one where the transmission probability of each layer was the same (homogeneity in transmission probabilities). As a result, we produced four distinct scenarios that can be classified as follows: no superspreading and homogeneity in transmission probabilities, superspreading and homogeneity in transmission probabilities, no superspreading and higher in-home transmission probability, and superspreading and higher in-home transmission probability. This allowed us to study the impact of household structure on the estimation of transmission parameters. Specifically, it is often assumed that the probability of transmission is higher and contact tracing is more complete relative to community contacts. Table 2 displays the parameters that we varied within Covasim to achieve the desired scenarios. Source code for these calibrations can be found in the linked repository (https://github.com/Henry-Bayly/Covasim-Paper-Code).

Each scenario was run 1,000 times. The secondary transmission chains were split into four groups corresponding to the contact layer in which the transmission occurred. The transmission chains corresponding to extra-household contacts were reduced by 25%, 50%, and 75%, while household contacts were completely observed. To reduce the ‘chain size’ we determined the total number of infections caused by a person and then multiplied that number by the corresponding percentage. Doing this represents the situation where only a portion of a person’s truly infected contacts were observed. The number of infections across all layers were aggregated to estimate R0 and k using maximum likelihood with model (2). We compared the estimates of R0 and k when all contact tracing and case data are observed to scenarios when this data is incomplete.

## Results

**Figure 1:**
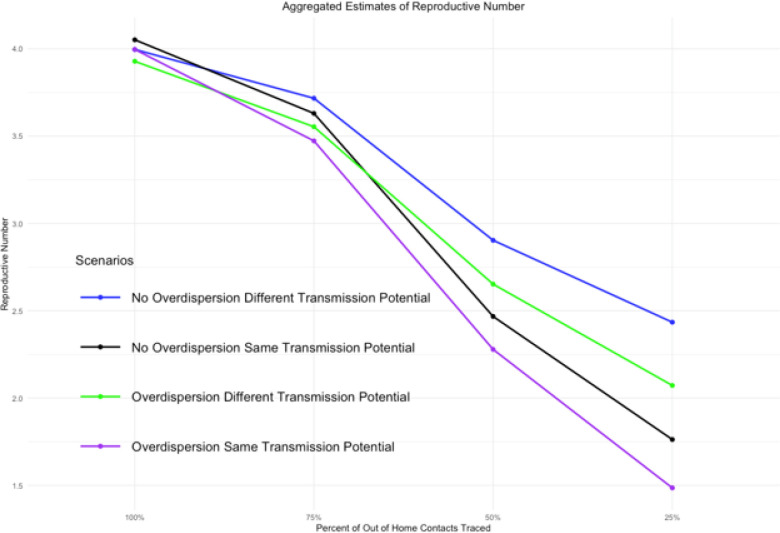
As the amount of extra-household contacts that are traced decreases our estimate of R0 decreases and thus becomes more inaccurate. We use the reference point of when 100% of extra household contacts are traced as our best achievable estimate and therefore our reference point.

In general, when the completeness of contact tracing data decreases for extra-household contacts, the estimate of $R_{0}$ decreases while k tends to increase. In the simplest scenario, where there is no superspreading and the transmission potential is the same for all contacts, the actual estimate of $R_{0}$ decreased from 4.05 when all contacts were observed to 1.76 when only 25% of extra-household contacts were traced. The bias was even more pronounced in this setting when superspreading was introduced with the estimated $R_{0}$ decreasing from 4.00 to 1.49. This pattern was the same in the scenarios with higher within-household transmission, but the bias was slightly less (Table 1, Fig. 1).

**Figure 2:**
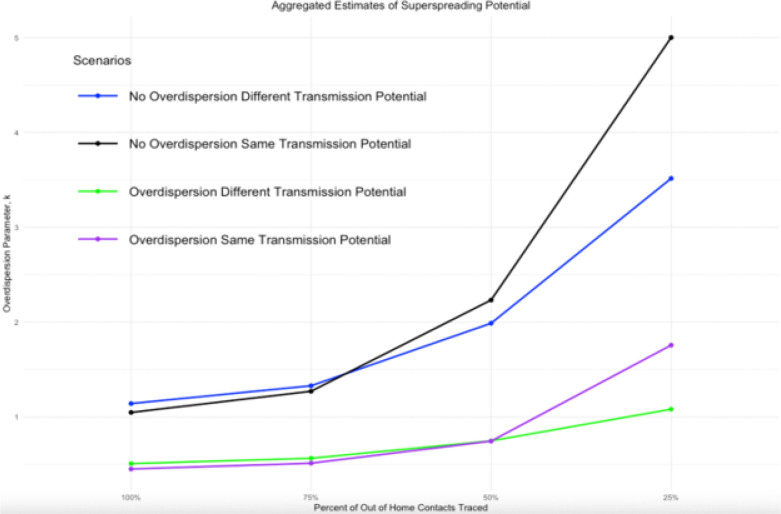
As the amount of extra-household contacts that are traced decreases our estimate of k increases and thus becomes more inaccurate. We use the reference point of when 100% of extra household contacts are traced as our best achievable estimate and therefore our reference point. When k increases past 1, we would conclude no presence of superspreading. This dispersion, then, would better be classified by something like Poisson transmission. *The estimate of k for the non-overdispersed and equal transmission potential by layer case is cutoff at 5.

In scenarios with superspreading behavior, if only 25% of extra-household contacts were traced, the estimated value of k increased from 0.45 to 1.75. In the case where there was equal transmission potential with all contacts and when household transmission was more likely, the estimated value of k increased from 0.51 to 1.08. This means that incomplete contact tracing generally resulted in estimates that would indicate no superspreading (Table 1, Fig. 2).

Interestingly, when we assume that the transmission potential is higher for household contacts than for other contacts, the biases in both $R_{0}$ and k are less pronounced. This behavior is consistent with our expectations because the estimate of $R_{0}$ is dependent on the total number of observed positive contacts. When we assume equal transmission in each layer, the distribution of contacts across each layer is equal. Therefore, reducing the number of contacts in the extra-household layers has a higher impact on the estimation of $R_{0}$. Whereas, when there is less transmission occurring in the extra-household layers compared to the within home layer, the number of contacts reduced is also less, allowing for a more complete estimate to be computed. While there is notably less bias in the estimates, the bias itself is still substantial.

## Discussion

In this paper we used an agent-based model to quantify the impact of incomplete and preferential contact tracing data on estimates of the basic reproductive number, , and superspreading potential as measured by the overdispersion parameter, k. Our results indicate that when contact tracing preferentially selects household contacts above other types of contacts, we are more likely to underestimate the reproductive number and the superspreading potential. In other words, it is possible to incorrectly conclude that there is no superspreading behavior when contact tracing data is incomplete and household contacts are preferentially traced. Incomplete contact tracing can also lead to artificially low estimates for the of a disease.

These results are of particular importance because contact tracing data was used early in the SARS-CoV-2 pandemic to infer trends in transmission and understand the superspreading potential of the disease [6,39,42]. These studies came from Indonesia[43], India[44], Hong Kong [39], Japan [42], Singapore [45], and mainland China [12] during the early phase of the pandemic. Some of the larger studies in Indonesia and India likely missed many contacts. The study in Japan largely focused on data that came from identified clusters, meaning that single cases were not included. The remaining studies were smaller and sampled a wider range of confirmed cases.

The Indonesian and Indian studies did not estimate the reproductive number using the contact tracing data but did use other techniques to estimate the reproductive number. The Indonesian study did use an SEIR type model to estimate a reproductive number to be 6.79 and 2.47 for the two locations they studied, and the Indian study estimated time-varying reproductive numbers ranging between 1.0 and 4.3. In the remaining studies, the estimated reproductive number using contact tracing data was less than one (range of estimates: 0.4–0.6). While the discrepancy between these estimates derived from contact tracing data might partially be explained by the strict control measures in place in these settings, our results would also indicate that incomplete contact tracing would result in underestimation of the reproductive number, indicating more transmission could have been occurring.

All these studies estimated the overdispersion parameter using contact tracing data and found evidence of a high degree of superspreading with overdispersion parameter estimates ranging from 0.06 in Indonesia to 0.58 in Shenzhen China. Our simulation results would imply that incomplete contact tracing would mean that the superspreading was even more extreme than that measured. The study in Japan, which largely focused on cases of clustered spread, excluding unlinked cases, estimated an overdispersion parameter of 0.22 (95% CI: 0.19–0.26) and the addition of singleton cases would likely reduce this value even more.

Since most real-world scenarios cannot provide complete data, we often rely on studies based on partial sampling of contact networks, for instance household contact studies. Here we show that doing so will always lead to biased estimates of the basic reproductive number, . We also show that this will tend to estimate less superspreading behavior, even when it exists. However, if all our data is coming from settings with high potential for transmission (i.e., dense group living settings, such as universities) or we are more likely to identify and contact trace those with larger contact networks, the bias might skew in the other direction, indicating more superspreading and transmission than is present in lower-contact settings. This implies that the data obtained from either setting will produce biased results if incomplete, but also if applied to a non-similar contact setting. Because of this, our study highlights the necessity of obtaining complete and accurate data to make inferences about the transmission potential of novel respiratory viruses and novel variants of SARS-CoV-2. Our study also suggests that the completeness of contact tracing should be considered when comparing competing estimates, as this can have a powerful impact on the estimated and k values. This finding is especially notable in the context of the fact that estimates for both these parameters have been quite inconsistent at times, even for the same variant [37–39]. While some of these differences may arise from regional variations in contact patterns, the implementation of contact tracing is another factor that may artefactually deflate estimates for the transmission potential of a respiratory disease.

The primary limitation of this study is that we do not fully explore mitigation measures that can limit extra-household transmission. An example of this was seen in many East Asian countries where significant restrictions were placed on an individual’s social mobility [26–30]. As a result, extra-household contacts were reduced drastically and the potential for superspreading-like behavior decreases. Our study assumes none of these social mobility restrictions are present. Future studies might be interested in incorporating these restrictions to understand their impacts on estimation of the superspreading potential as opposed to strictly biological constraints on superspreading potential. Furthermore, we implemented this model and used estimates solely for SARS-CoV-2, and thus the generalization of these results to other diseases may not be appropriate. However, there is no reason to think that the trends should be any different for a person-to-person transmissible respiratory disease.

Any study that relies on contact tracing data to make epidemiological inferences about transmission must be sensitive to the potential incompleteness of this data. This incompleteness can arise due to recall bias, an incomplete understanding of who qualifies as a contact, an inability to identify contacts who are not well-known to the index case, and incomplete public health infrastructure. The results of this study make ever more apparent the need for a complete public health response to SARS-CoV-2 and other person-to-person transmissible diseases. Complete in this context includes public health officials efficiently and effectively communicating grounded and interpretable truths regarding the transmission of the diseases. However, complete in this case also refers to the public’s response and willingness to ‘buy in’ to the most effective strategies to inhibit the flow of future diseases. The larger the disconnect between these two, the larger our gap will be to understanding the truths of the disease that allow us to effectively mitigate its spread and impact.

## Figures and Tables

**Table 1:** Estimates of R0 and k from the simulations using Covasim. Four scenarios are shown varying the amount of superspreading and whether there is higher transmission potential for household contacts relative to other contacts. Within each scenario, we consider varying degrees to which extra-household contacts are traced. Estimates shown are the mean, min, and max values of the simulations results.

	Estimate of R0 (Min, Max)	Estimate of k (Min, Max)
**Superspreading Behavior and Higher Household Transmission Potential**		
Perfect Contact Tracing	3.93 (3.81, 4.03)	0.51 (0.47, 0.55)
75% Traced Out of Home	3.55 (3.44, 3.65)	0.57 (0.52, 0.61)
50% Traced Out of Home	2.65 (2.57, 2.73)	0.75 (0.69, 0.81)
25% Traced Out of Home	2.07 (1.99, 2.15)	1.08 (0.98, 1.20)
**No Superspreading Behavior and Higher Household Transmission Potential**		
Perfect Contact Tracing	4.00 (3.91, 4.06)	1.14 (1.06, 1.22)
75% Traced Out of Home	3.72 (3.64, 3.77)	1.33 (1.23, 1.42)
50% Traced Out of Home	2.90 (2.84, 2.96)	1.99 (1.79, 2.19)
25% Traced Out of Home	2.43 (2.36, 2.50)	3.52 (2.75, 4.25)
**Superspreading Behavior and Equal Transmission Potential By Layer**		
Perfect Contact Tracing	4.00 (3.86, 4.08)	0.45 (0.42, 0.48)
75% Traced Out of Home	3.47 (3.36, 3.54)	0.51 (0.47, 0.55)
50% Traced Out of Home	2.28 (2.20, 2.33)	0.75 (0.68, 0.80)
25% Traced Out of Home	1.49 (1.42, 1.53)	1.76 (1.51, 2.06)
**No Superspreading Behavior and Equal Transmission Potential By Layer**		
Perfect Contact Tracing	4.05 (3.97, 4.12)	1.05 (0.98, 1.12)
75% Traced Out of Home	3.63 (3.56, 3.69)	1.27 (1.18, 1.37)
50% Traced Out of Home	2.47 (2.42, 2.51)	2.23 (2.03, 2.45)
25% Traced Out of Home	1.76 (1.72, 1.81)	35.08 (12.62, 162.61)

**Table 2:** Covasim parameters varied to achieve the desired scenarios. The population type in all scenarios was set to Hybrid. Transmissibility by layer refers to the infectiousness of contacts at each layer of a person’s contact network (home, school, community, and work). Therefore, all=0.6, means that the values for the infectiousness are set to 0.6 for all the contact layers. On the other hand, Default uses the normal values provided by Covasim. The global transmissibility distribution describes the overall transmission potential of the virus.

Simulation Type	Transmissibility by Layer	Global Transmissibility Distribution	Population Size
Superspreading Behavior and Equal Transmission Potential By Layer	All = 0.6	Negative Binomial Parameter 1 = 100 Parameter 2 = 0.5	5,000
No Superspreading Behavior and Equal Transmission Potential By Layer	All = 0.6	Negative Binomial Parameter 1 = 100 Parameter 2 = 2.0	5,000
Superspreading Behavior and Higher Household Transmission Potential	Default	Negative Binomial Parameter 1 = 100 Parameter 2 = 0.5	5,000
No Superspreading Behavior and Higher Household Transmission Potential	Default	Negative Binomial Parameter 1 = 100 Parameter 2 = 2.0	5,000

## Data Availability

All data and code are available at (https://github.com/Henry-Bayly/Covasim-Paper-Code).
